# Sustained Improvement in Paediatric ENT Emergency Referral Accuracy Through a Multimodal Educational Strategy: A Longitudinal Study

**DOI:** 10.7759/cureus.100306

**Published:** 2025-12-29

**Authors:** Raul Simon, Matija Daniel

**Affiliations:** 1 ENT Department, Nottingham University Hospitals NHS Trust, Nottingham, GBR; 2 School of Medicine, University of Nottingham, Nottingham, GBR

**Keywords:** educational campaign, ent emergency clinic, guideline adherence, paediatric ent, quality improvement study

## Abstract

Introduction: The ENT emergency clinics often rely on SHO (Senior House Officer) level clinicians, such as Foundation Doctors and Core Trainees, to triage and assess urgent referrals. Emergency clinics receive urgent referrals for both adult and paediatric patients. Inconsistent adherence to locally available referral guidelines, which indicate what is appropriate for emergency clinic booking and what is not, can lead to inappropriate clinic utilisation, resulting in inefficiency and suboptimal patient care. This study aimed to evaluate guideline adherence in paediatric-only referrals to an SHO-led ENT emergency clinic in a tertiary centre and determine whether simple, low-cost interventions could improve and sustain compliance.

Methods: This study was a six-cycle Quality Improvement (QI) project. All paediatric patients seen in an SHO-led ENT emergency clinic between December 2020 and November 2022 at Queen’s Medical Centre, Nottingham, UK, were analysed. A total of 209 encounters were reviewed using the Trust’s electronic record system. Referral reasons were compared with the local Trust paediatric ENT emergency clinic referral guidelines. The PDSA (Plan Do Study Act) framework for QI was applied, and following the baseline audit, interventions included targeted junior doctor teaching, dissemination of referral criteria via online communication platforms, and a poster campaign in key clinical areas.

Results: Baseline adherence was 38.5% compared with a benchmark standard of 100% adherence. Following educational and communication-based interventions, the proportion of guideline-compliant referrals increased to 74.0%. This improvement achieved statistical significance and was sustained across subsequent audit cycles despite frequent SHO turnover. Staff demonstrated the strongest adherence in referrals for foreign bodies and epistaxis. Common inappropriate referrals included cerumen impaction and a discharging ear.

Conclusions: Simple and sustainable interventions, including structured teaching, digital dissemination, and poster-based prompts, significantly improved guideline adherence in an SHO-led paediatric ENT emergency clinic. This low-cost QI model is easily reproducible in similar healthcare settings.

## Introduction

Paediatric ENT emergencies present unique challenges in triage and referral. To manage the demand of paediatric emergency presentations that do not have to be seen immediately (for example, epistaxis that settles, non-battery foreign bodies in the ear), many hospitals across the UK operate ENT emergency clinics (for both adult and paediatric patients) led by senior house officers (SHOs) under consultant or registrar support [1]. This allows patients access to timely ENT input, without having to wait for routine consultant-led clinic appointments. The ENT SHOs have training in the management of ENT emergencies, but lack the breadth of experience that a senior doctor (registrar or consultant) would possess [2]. It is therefore crucial that patients booked at the emergency clinics have conditions that the SHO is trained to manage. Despite this, recent literature shows that a significant proportion of referrals to the SHO-led emergency clinic are not appropriate [3]. Furthermore, to preserve timely access to this service, it is important that only genuine emergencies are seen.

Research shows that educational interventions and referral toolkits have a positive impact on the ENT junior doctor’s confidence and preparedness for on-call clinical duties [4]. Our unit has consequently developed comprehensive guidelines on which emergency presentations can be seen in the SHO-led emergency clinic and which require more senior input. The guidelines also specify the urgency with which common presentations should be managed. This ensures that the patient sees the right person the first time and ensures equity of waiting times across patient populations.

However, frequent SHO rotation and variable ENT experience in a busy on-call environment with sometimes overwhelming clinical responsibilities often result in poor awareness of and compliance with the referral criteria [5,6]. It is worth noting that the main gatekeepers for referring to the emergency clinic are SHOs themselves when they receive phone calls from general practitioners (GPs) or accident and emergency (A&E) doctors during their on-calls and triage patients by streamlining the referral into an on-call review, emergency clinic booking (typically within a few days to a week), or a routine outpatient clinic. Hence, the referral guidelines are mostly utilised by this cohort of clinicians, who consequently become important stakeholders in efficient resource allocation and delivering optimal patient care. This is reflective not only of ENT practice, but also of a cross-speciality situation in which SHO-level doctors are key stakeholders in the referral process [7,8]. This article was presented as an oral abstract at the 16th Congress of the European Society of Paediatric Otorhinolaryngology in Liverpool, United Kingdom, on May 22, 2023.

## Materials and methods

We conducted a six-cycle Quality Improvement (QI) study, utilizing the PDSA (Plan-Do-Study-Act) methodology, in an SHO-led paediatric ENT emergency clinic at a UK tertiary centre teaching hospital. The aims were to measure the proportion of guideline-appropriate referrals, improve adherence using simple and low-cost interventions, and sustain improvements across rotations despite changes in SHO staff.

Study rationale

There was a perceived increase in patients who were inappropriately booked to the paediatric ENT emergency clinic, resulting in wasted clinic slots, inappropriately managed patients, or increased senior input.

Setting

The study took place in the paediatric ENT emergency clinic at a UK tertiary centre teaching hospital, Queen’s Medical Centre (QMC) in Nottingham, Nottingham University Hospitals NHS Trust (NUH). The clinic is SHO-led with consultant or registrar supervision, and referrals are mainly managed by on-call SHOs who act as gatekeepers.

Benchmark standards

Local NUH guidelines were developed by local ENT consultants. The guidelines highlight red flags where certain conditions render referrals inappropriate for an SHO-led emergency clinic, as these need to be managed on-call, such as severe epistaxis, battery foreign bodies, and airway concerns. Likewise, some conditions are deemed inappropriate for the emergency clinic as they are best managed in elective clinics, for example, simple cerumen or recurrent otitis media. Lastly, the guidelines specify the conditions under which certain presenting complaints or diagnoses are appropriate for an SHO-led emergency clinic, for example, otitis externa requiring a pope wick insertion or removal. The benchmark standard for comparison of current practice was proposed as 100% guideline adherence, stipulating that with guidelines present, all conditions referred should be triaged and streamlined appropriately. An excerpt from the guideline is available in Table 1.

**Table 1:** Excerpt from the departmental guidelines on paediatric ENT emergency referrals The guideline directs the clinician who is accepting the referral into appropriate action regarding the referral with several different outcomes, such as ENT to see on call, ENT to see within 24 hours, or to book into the ENT emergency clinic. ED: emergency department, GP: general practitioner, ENT: ear, nose and throat, NBM: nil by mouth, NUH: Nottingham University Hospitals, SHO: senior house officer

Site	Condition	Watch for	ED action	GP action	ENT on call	ENT within 24 hours	Emergency clinic	Routine clinic	Notes
Ear	Foreign body	BATTERIES: see immediately. LIVE INSECTS: kill with olive oil and see on call	Phone ENT on call	Phone ENT on call	If battery	Not appropriate	Yes	Not appropriate	
	Otitis externa: usually in teenagers, swollen ear canal, tragal tenderness	PINNA CELLULITIS: see on call	Analgesia. Treat with antibiotic/steroid drops. Keep dry and no cotton buds. If that is not effective, or the ear canal is closed, then phone ENT SHO. Consider a routine clinic referral if recurrent	Analgesia. Treat with antibiotic/steroid drops. Keep dry and no cotton buds. If that is not effective, or the ear canal is closed, then phone ENT SHO. Consider a routine clinic referral if recurrent	If pinna cellulitis	See as an emergency attender	Not usually, unless needs wick removal	Yes, for follow-up, or if recurrent. Can also use a nurse-led clinic	Otitis externa: tragus tender, ear canal closed. Otitis media: canal filled with discharge but not closed, discharge often pulsatile. Otitis media: younger children. Externa: teenagers
	MASTOIDITIS. Pinna displaced, redness, and swelling over the mastoid	INTRACRANIAL COMPLICATION	Phone ENT on call	Phone ENT on call	Admit. Consult the NUH guideline. NBM until senior review	Not appropriate	Not appropriate	For follow-up after admission	Keep NBM until review
	Ear discharge in a child under 8, otitis media, infected perforated eardrum, discharging grommets	MASTOIDITIS, INTRACRANIAL COMPLICATION: see on call	Treat with antibiotic drops. If already had drops, swab for micro and change drops to a different antibiotic. ENT clinic referral via letter	Treat with antibiotic drops. If already had drops, swab for micro and change drops to a different antibiotic. ENT clinic referral via letter or C+B	See if there is a complication. Advise referrer to use drops (cipro or aminoglycoside – discuss risk). Swab if not settling after drops. Water precautions	Not appropriate	Not appropriate. If the first-line drops are not effective, GP should swab and adjust antibiotics based on micro	Yes	Otitis externa: tragus tender, ear canal closed. otitis media: canal filled with discharge but not closed, discharge often pulsatile. Otitis media: younger children. Externa: teenagers

Study design

Six audit cycles were analysed from December 2020 to November 2022 at approximately four to six-month intervals. The first three cycles followed the PDSA methodology, whereby a new intervention was introduced in each subsequent cycle, whereas the latter three cycles served as a continuous assessment of the sustainability of existing interventions implemented in the first three cycles. No new interventions were implemented in the latter three cycles. Data from paediatric patients were collected from the Trust’s electronic patient record. Parameters collected included age, referral reason, and adherence to guidelines, dichotomised into a simple yes or no based on whether the referral was appropriate according to the guidelines. Statistical significance was assessed using the chi-square test to compare guideline compliance at baseline (first cycle) with the combined post-intervention period (cycles 2-6), and to compare guideline adherence for conditions with the greatest increase in compliance between the first and the last cycle. A p-value <0.05 was considered statistically significant. Data analysis was performed using Microsoft Excel version 2024 (Microsoft Corporation, Redmond, Washington).

Intervention 1

Teaching sessions for junior doctors were conducted, typically during local monthly Governance meetings, alongside digital dissemination of guidance via online communication platforms (WhatsApp groups). During these sessions, SHOs were informed that the guidelines exist and were shown how using them could improve efficiency by enabling rapid assessment of whether a condition was appropriate for referral to the emergency clinic. The guidelines supported on-call decision-making.

Intervention 2

**Figure 1:**
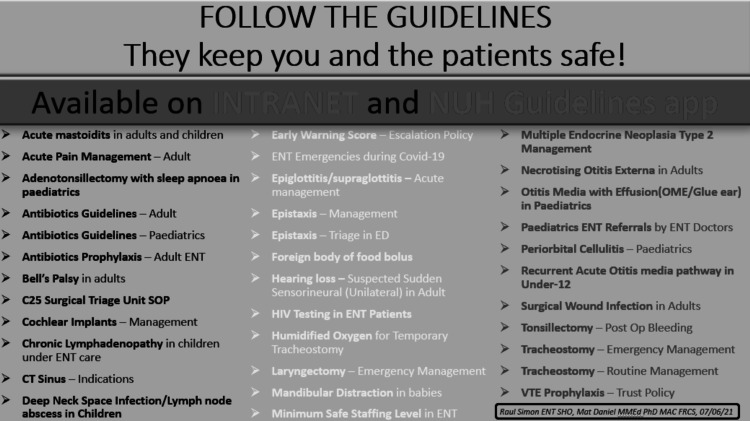
Poster reinforcing guideline adherence Several copies of this poster were disseminated throughout key clinical areas to improve awareness of, and aid compliance with, pertinent guidelines. The poster serves as a reminder for all relevant ENT departmental guidelines, not just the paediatric ENT emergency clinic referral guidelines, thus broadening the scope of the intervention. Reproduced with permission from Nottingham University Hospitals NHS Trust.

A poster campaign was implemented to reinforce referral criteria and guideline awareness in key clinical areas. The poster used is shown in Figure 1. Posters were displayed in clinical areas such as wards and clinic offices to increase exposure among SHOs, demonstrating the range of guidelines available to the SHO cohort in everyday clinical practice and highlighting their importance.

Intervention 3

Continuous verbal reinforcement of the importance of guidelines, particularly to incoming SHOs, was provided by the authors of the QI study. This intervention was actively utilised across all six cycles.

Outcome measure

The proportion of referrals deemed guideline-compliant before and after the intervention was measured over time.

## Results

In total, there were 209 referrals to the paediatric ENT emergency clinic across the six cycles (two years). The most common referral reasons were epistaxis, foreign body, impacted wax, and acute otitis media (AOM) with ear discharge.

Baseline guideline compliance was 38.5% following completion of the first cycle. After the interventions described in the Methods section, peak compliance rose to 76.5% in the second cycle and ultimately plateaued at 74% in the sixth cycle. This represented a near doubling of appropriate referrals over the two-year period and achieved statistical significance (p = 0.006). This demonstrated sustained improvement across subsequent cycles despite SHO rotation and learning curves among new cohorts.

**Figure 2:**
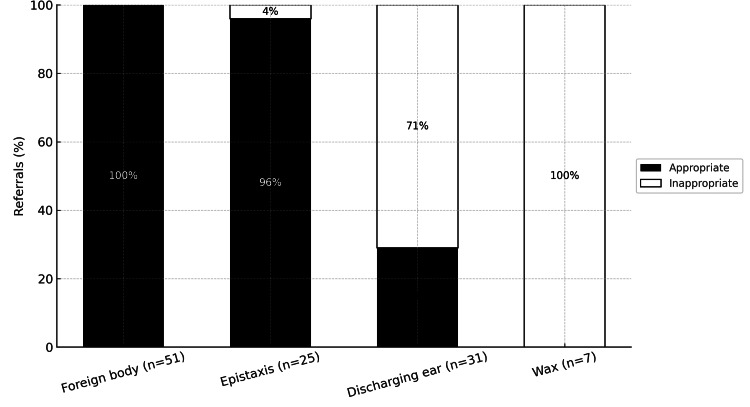
Distribution of referral types seen across audit cycles, indicating their guideline-compliance status The most common conditions which were appropriately referred to the paediatric ENT emergency clinic were foreign body and epistaxis with discharging ear and cerumen impaction being the most common inappropriately referred conditions.

The strongest compliance with referral guidelines was observed for foreign bodies and epistaxis, as shown in Figure 2. The most common inappropriate referrals were cerumen impaction and a discharging ear.

**Figure 3:**
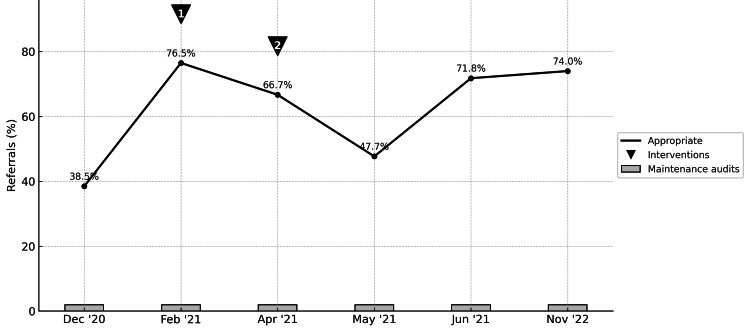
Percentage of appropriate referrals to the paediatric ENT emergency clinic (December 2020 to November 2022) Compliance over time (two years and six audit cycles) demonstrates sustainability. The timing of introducing specific tools for implementing change is noted via superimposed triangles. The dip in the fourth cycle could potentially be explained by a temporary fatigue of efforts in the educational campaign. Intervention number one was the educational campaign via online platforms introduced in the second cycle,  and Intervention number two was the poster campaign introduced in the third cycle. Intervention number three (verbal reinforcement) has been continuously and consistently used throughout all cycles.

Compliance levels across all six cycles clearly demonstrate sustainability, as illustrated in Figure 3. The timing of implementation of specific tools for change is also shown in the figure.

**Figure 4:**
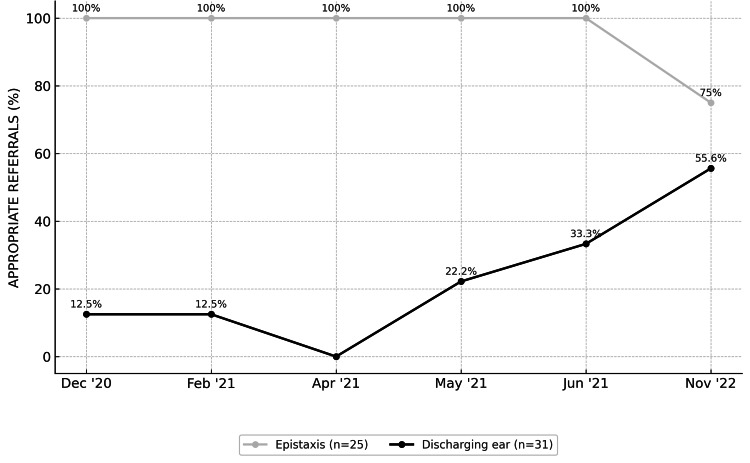
Quality improvement impact on different conditions across the six cycles This graph highlights the strengths of the SHO cohort with a sustained high level of guideline adherence and appropriate management of epistaxis, whilst simultaneously showcasing areas of the greatest impact of the QI study interventions, most notably the discharging ear, which was initially one of the most commonly inappropriately referred (hence, guideline non-compliant) conditions, and where guideline adherence (and management appropriateness) gradually increased to more than fourfold following the interventions implemented. SHO: Senior House Officer, QI: Quality Improvement.

An alternative method used to assess the impact of the QI study was evaluation of guideline compliance for specific conditions. Three benchmarks were selected: conditions that were most consistently guideline-compliant (epistaxis), representing SHO-level strengths; conditions that were most consistently guideline-noncompliant (impacted wax), highlighting areas for improvement; and the condition with the greatest improvement in compliance attributable to the QI study (discharging ear, AOM with tympanic membrane perforation). These findings are presented in Figure 4. The increase in guideline adherence for discharging ear in the final cycle did not reach statistical significance (p = 0.170), despite a fourfold increase.

## Discussion

This QI study shows substantial, statistically significant, and sustained improvement in guideline adherence in an SHO-led paediatric ENT emergency clinic in a tertiary centre teaching hospital. Digital dissemination, visual prompts, and targeted teaching collectively enhanced junior doctor decision-making. Sustained improvement despite SHO turnover demonstrates the durability and effectiveness of the intervention model, confirming that changes were embedded at the system level rather than dependent on individuals. The low-cost solutions implemented also underline the cost-effectiveness of this strategy, a crucial factor within the confines of the ENT resource-limited environment pertinent to the NHS [9].

An important learning point is the clear value of multimodal reinforcement. Teaching alone produced an initial improvement, but ongoing accessibility of information, particularly via online platforms and strategically placed posters, ensured that correct referral pathways were always available "at the point of decision". This reduced cognitive load for SHOs who often make triage decisions under time pressure. The audit also highlighted areas in which junior doctors' confidence and knowledge were strongest (epistaxis, foreign bodies) versus those requiring further educational focus (AOM with TM perforation). This creates an opportunity for targeted induction teaching and simulation-based training to strengthen weaker areas. Although the increase in guideline compliance for the discharging ear did not reach statistical significance, the fourfold increase remains considerable as a potential impact of the QI methodology.

The limitations of the study include the single-centre design, which limits generalisability, and the observational methodology, which limits causal inference and implies reliance on routinely collected data that may be incomplete or misclassified. As current data were unavailable at the time of analysis, the long-term impact of the study is not well represented. In addition, the PDSA methodology was not applied consistently across all six cycles, limiting reproducibility, and no patient outcome measures were collected or analysed. Nevertheless, the QI framework demonstrated how continuous feedback loops and real-time visibility of performance can motivate behavioural change. Displaying compliance trends and highlighting improvements fostered ownership among SHOs and helped maintain momentum. Finally, the project illustrates how empowering junior clinicians through involvement in QI can enhance both service quality and trainee engagement. Collectively, these findings suggest that similar SHO-led clinics across the UK could benefit from adopting this QI model.

## Conclusions

Despite pertinent limitations, this quality improvement study demonstrates that a low-cost, multimodal educational and communication strategy can produce a substantial and sustained improvement in the appropriateness of referrals to an SHO-led paediatric ENT emergency clinic. The multimodal design of the intervention (teaching sessions, digital dissemination, posters, and continuous reinforcement) ensured that guidance remained easily accessible across multiple points in the referral pathway. Importantly, the methods used in this project were intentionally simple, cost-neutral, and fully integrated into existing departmental structures. This means the model is highly scalable and reproducible, not only across ENT departments within the UK but also in other specialities where early career (SHO-level) doctors act as primary gatekeepers for emergency referrals.

Acknowledging the study limitations, the findings support the broader conclusion that sustainable improvements in clinical triage behaviour can be achieved without major financial investment or structural change, provided that interventions are context-specific, frequently reinforced, and embedded into routine communication channels. As pressures on emergency services and specialist clinics continue to increase, this approach offers a pragmatic, effective, and easily adoptable framework for improving patient flow, resource utilisation, and quality of care.
